# Supplementary material for: Multidisciplinary care for opioid dose reduction in patients with chronic non-cancer pain: A systematic realist review
Source: PLoS One. 2020 Jul 27;15(7):e0236419. doi: 10.1371/journal.pone.0236419 (PMC7384622; doi:10.1371/journal.pone.0236419)
Supplement: S1 Table — (DOCX) [file pone.0236419.s001.docx]

|  |
| --- |
| **Search Strategy** |
| 1 exp Analgesics, Opioid/ (105348)  2 Opioid-Related Disorders/ (11760)  3 exp Morphine Derivatives/ (48629)  4 opiate$.tw,kf. (24092)  5 opioid$.tw,kf. (76869)  6 acemethadone.tw,kf,nm. (0)  7 acetylmethadol.tw,kf,nm. (266)  8 alfentanil.tw,kf,nm. (2358)  9 alphaprodine.tw,kf,nm. (182)  10 anileridine.tw,kf,nm. (60)  11 benzomorphan$.tw,kf,nm. (974)  12 buprenorphine.tw,kf,nm. (6546)  13 butorphanol.tw,kf,nm. (1537)  14 carfentanil.tw,kf,nm. (295)  15 codeine.tw,kf,nm. (6580)  16 dextromoramide.tw,kf,nm. (408)  17 dextropropoxyphene.tw,kf,nm. (1647)  18 dezocine.tw,kf,nm. (138)  19 diacetyl morphine.tw,kf,nm. (10)  20 diamorphine.tw,kf,nm. (454)  21 dihydroetorphine.tw,kf,nm. (70)  22 dimepheptanol.tw,kf,nm. (0)  23 dionine.tw,kf,nm. (34)  24 diphenoxylate.tw,kf,nm. (376)  25 diprenorphine.tw,kf,nm. (742)  26 dihydrocodein$.tw,kf,nm. (517)  27 dihydrohydroxycodeinone.tw,kf,nm. (4)  28 dihydromorphine.tw,kf,nm. (419)  29 dihydromorphinone.tw,kf,nm. (109)  30 dipipanone.tw,kf,nm. (36)  31 dynorphin.tw,kf,nm. (4016)  32 endomorphin.tw,kf,nm. (740)  33 enkephalin$.tw,kf,nm. (20929)  34 eseroline.tw,kf,nm. (48)  35 etorphine.tw,kf,nm. (1077)  36 ethylketocyclazocine.tw,kf,nm. (804)  37 ethylmorphine.tw,kf,nm. (951)  38 fenoperidine.tw,kf,nm. (0)  39 fentanyl.tw,kf,nm. (20913)  40 hydrocodon$.tw,kf,nm. (1080)  41 hydromorphon$.tw,kf,nm. (1927)  42 hydroxycodeinone.tw,kf,nm. (17)  43 isocodeine.tw,kf,nm. (5)  44 isonipecain.tw,kf,nm. (0)  45 isopromedol.tw,kf,nm. (7)  46 kaolin-pectin.tw,kf,nm. (30)  47 ketobemidone.tw,kf,nm. (174)  48 levallorphan.tw,kf,nm. (501)  49 levodroman.tw,kf,nm. (0)  50 levomethadryl.tw,kf,nm. (0)  51 levorphan$.tw,kf,nm. (837)  52 meperidine.tw,kf,nm. (6678)  53 meptazinol.tw,kf,nm. (229)  54 methadol.tw,kf,nm. (63)  55 methadone.tw,kf,nm. (15642)  56 methadyl acetate.tw,kf,nm. (414)  57 morphia.tw,kf,nm. (52)  58 morphine.tw,kf,nm. (54714)  59 methynaloxone.tw,kf,nm. (0)  60 nalbuphine.tw,kf,nm. (945)  61 nocistatin.tw,kf,nm. (107)  62 opium.tw,kf,nm. (3414)  63 oxycodein$.tw,kf,nm. (3)  64 oxycodone.tw,kf,nm. (3343)  65 oxymorph$.tw,kf,nm. (776)  66 pantopon.tw,kf,nm. (9)  67 papaveretum.tw,kf,nm. (138)  68 paracymethadol.tw,kf,nm. (28)  69 paregoric.tw,kf,nm. (56)  70 pentazocine.tw,kf,nm. (2988)  71 pethidine.tw,kf,nm. (2329)  72 phenazocine.tw,kf,nm. (531)  73 phenbenzorphan.tw,kf,nm. (0)  74 phenethylazocine.tw,kf,nm. (0)  75 phenoperidine.tw,kf,nm. (263)  76 pirinitramide.tw,kf,nm. (259)  77 promedol$.tw,kf,nm. (270)  78 propoxyphene.tw,kf,nm. (910)  79 protopine.tw,kf,nm. (249)  80 pyrrolamidol.tw,kf,nm. (18)  81 remifentanil.tw,kf,nm. (4675)  82 sufentanil.tw,kf,nm. (2694)  83 talwin.tw,kf,nm. (57)  84 tapentadol.tw,kf,nm. (416)  85 thebaine.tw,kf,nm. (520)  86 theocodin.tw,kf,nm. (0)  87 tilidine.tw,kf,nm. (180)  88 tramadol.tw,kf,nm. (4739)  89 trimeperidine.tw,kf,nm. (26)  90 Clonidine/ (13021)  91 clonidine.tw,kf,nm. (17837)  92 or/1-91 [opioid concept] (211076)  93 exp Pain/ (362431)  94 exp Complex Regional Pain Syndromes/ (5269)  95 exp Neuralgia/ (18188)  96 Pain Measurement/ (77982)  97 pain.tw,kf. (551499)  98 or/93-97 [Pain concept] (710671)  99 (taper* or wean* or detox* or withdraw* or discontinu* or tolerance or conversion or cessation or substitution).tw,kf. (867331)  100 ((dos* or opioid* or opiate*) adj3 (reduc* or cessation or ceas* or stop*)).tw,kf. (55897)  101 or/99-100 [Dose reduction concept] (914655)  102 exp Therapeutics/ (4199904)  103 exp Psychotherapy/ (181821)  104 exp Rehabilitation Centers/ (13753)  105 exp Health Personnel/ (465372)  106 ((cognit* or behavio?r* or family or psychosocial*) adj5 (therap* or intervention*)).tw,kf. (76041)  107 cbt.tw,kf. (9021)  108 ((animal* or pet* or art or alternative or combin* or complement* or implosive or relax* or colo?r or dance or emotion* or exercise or gestalt or horticultural or laughter or music or narrative or occupational or person* or play or psychoanaly* or physical or recreation or reality or relaxation or behav* or socioenvironment* or spiritual*) adj3 therap*).tw,kf. (215676)  109 (aromatherap* or acupuncture* or biofeedback or neurofeedback or mindfulness or yoga or tai ji or tai chi or meditat* or hypnotherap* or hypnos* or bibliotherap* or psychotherap* or psychophysiol* or physiotherap* or therapeutic touch* or naturopath* or pray* or organotherap* or phytotherap* or reflexotherap*or prolotherap*).tw,kf. (126812)  110 exp Counseling/ (40682)  111 (counsel* or cope or coping).tw,kf. (166589)  112 Opiate Substitution Treatment/ (2238)  113 (diprenophine or nalmefene or nalorphine or naloxone or naltrexone or methadone or buprenorphine or clonidine or lofexidine or guanfacine or marijuana).tw,kf. (69180)  114 or/102-113 [non-pharmacologic & pharmacologic therapies] (5015088)  115 exp Patient Care Team/ (63887)  116 Pain management/ (28519)  117 Pain Clinics/ (1370)  118 (multidisciplinary or multi-disciplinary or MDC).tw,kf. (77817)  119 ((interdisciplinary or inter-disciplinary or interprofessional or inter-professional or collab* or integrat*) adj4 (collab* or care or team* or approach* or program* or model* or communicat* or rehab* or treatment* or clinic* or center? or centre?)).tw,kf. (214386)  120 (pain adj3 (center? or centre? or rehab? or clinic? or program* or management)).tw,kf. (32262)  121 (continuum adj2 care).tw,kf. (3240)  122 (team* adj3 care).tw,kf. (15206)  123 exp "delivery of health care, integrated"/ or exp patient care team/ (74091)  124 (IPC or MRP or CPRP?).tw,kf. (7849)  125 (multimodal* adj2 treatment).tw,kf. (5701)  126 or/115-125 [multidisciplinary care concept] (406768)  127 114 or 126 [expanded MDC concept] (5251584)  128 126 or 127 (5251584)  129 92 and 98 and 101 and 127 [opioid concept + pain concept + dose reduction concept + expanded MDC concept] (6350)  130 exp animals/ not humans.sh. [to remove animal-only studies] (4490575)  131 129 not 130 [opioid concept + pain concept + dose reduction concept + expanded MDC concept -- most animal studies removed] (4653) |
