# Supplementary material for: Multidisciplinary care for opioid dose reduction in patients with chronic non-cancer pain: A systematic realist review
Source: PLoS One. 2020 Jul 27;15(7):e0236419. doi: 10.1371/journal.pone.0236419 (PMC7384622; doi:10.1371/journal.pone.0236419)
Supplement: Attachment — Submitted filename: Response to Reviewers.docx [file pone.0236419.s003.docx]

**Response to Reviewers**

Manuscript: *Multidisciplinary care for opioid dose reduction in patients with chronic non-cancer pain: A systematic realist review*

| **Reviewer Comment** | **Response** | **Location of edits** |
| --- | --- | --- |
| **Reviewer #1** | | |
| Line 51-52, "Unintentional drug overdose deaths are high and on the rise throughout the Global North. The United..." --> Please define Global North for this article | Clarified as “the developed economies of North America, Australia and several European countries” | Page 2, Lines 51-52 |
| Line 55-56, "One large socioeconomic effect of these epidemics is that life expectancies in the US and Canada have dropped over the last several years -->Please re-word to remove "dropped", perhaps say "decreased" | Change made | Page 2, Line 57 |
| Line 78-79 "for tapering, or dose reduction. The Canadian guidelines provide a recommendation based on weak evidence --> Please better define or elaborate on "weak" | Quoted GRADE definition included in Canadian guidelines of “weak” and “strong” recommendations | Page 3, Lines 79-81 |
| Line 139-140 "Grey literature was searched from a variety of sources including Ovid EMBASE (conference proceedings and meeting abstracts), -->Define "grey" | Grey literature defined as “reports and information not published commercially” as per the Canadian Agency for Drugs and Technology tool that is cited. Also clarified that the Ovid EMBASE, etc. searches were targeted database searches rather than part of the grey literature search | Page 5, Lines 141-144 |
| Line 183-184 "A data extraction form was created in Excel and tested by multiple reviewers. Once validated, the form was replicated in Covidence and data extraction was conducted singly. -->How many reviewers? how was it validated? | Clarified the extraction piloting and validation process | Page 8, Lines 198-200 |
| Line 367-368,"On the contrary, in American programs, where there are cultural and political concerns about opioid use, there were" -->please elaborate on "political concerns" | Added the phrase “including in the form of significant national and regional opioid-related policies such as the declaration of a public health emergency by the Department of Health and Human Services” and citation | Page 22, Lines 389-391 |
| Line 476-477, "Interdisciplinary pain programs (IPPs) represent one of the best alternatives." --> Throughout this paper the focus is on multidisciplinary, rather than interdisciplinary, approach to pain and opioid reduction. These are two different models and approaches to pain care, both with pros and cons. This article should specifically define these two, and explain why multidisciplinary versus interdisciplinary was chosen as the methodological approach. | We have further clarified our definition of multidisciplinary care which aimed to hew closely to the description provided in the Canadian guidelines and definitions from previous reviews. We have clarified that this definition of MDC differs slightly from that of the IASP (<https://www.iasp-pain.org/PublicationsNews/NewsDetail.aspx?ItemNumber=6981>)  in that it is meant to be inclusive of interdisciplinary care.  We have also acknowledged, based on our previous clinical and research experience, that are a diversity of definitions and uses of the terms interprofessional, multidisciplinary, interdisciplinary, etc. | Pages 7, Lines 176-178 |
| Lines 543-546 "This could be operationalized in many ways, such as modified shared-care models which have been widely deployed to improve primary care access and capacity for mental health in Canada [134]. Likewise, persistent imbalances in institutional support (such as funding) --> The authors should add some discussion about (1)payment reimbursement models, single payer systems versus private insurance payers from region to region, and how this might impact opioid tapering as well as patient and provider resources, treatments, incentives, and patient outcomes. In the US, there is a lack of incentive in this regard, thus discussing this for the US and other populations would be important as well as (2) lack of pain related education in medical schools and residencies which could also impact opioid tapering in various populations | We have added a comment that strategies need to accommodate health system differences including, as example, reimbursement models and the role the primary versus tertiary care. We did not find any clear patterns regarding differences between programs that we could attribute specifically to payment models. For example, though Canadian *medical* care has universal access and is reimbursed through a single payer, this system typically *excludes* the non-medical services (such as psychologists and physiotherapists) which typify MDC programs in this area. We agree that such an analysis is very much worthwhile but is beyond the scope of this study.  We do agree that medical education throughout the continuum has an important role to play in improving opioid prescribing practices, and some of our team members lead the development, delivery and evaluation of such programs. Though there was not evidence of this gathered from this review, we have already noted that behaviour change strategies focused on prescribers, including education, *could* be effective in achieving the goals of long-term opioid dose reduction. Based on the findings of our current systematic review (in process) of continuing medical education programs for opioid prescribing, we are not aware of any programs that have successfully achieved long-term opioid dose reduction and so have only mentioned such a strategy provisionally. | Page 30, Lines 579-582 |
| **Reviewer #2** | | |
| "Multidisciplinary care for opioid dose reduction in patients with chronic non-cancer pain: A systematic realist review". Overall, the manuscript is very well-written. It is also generally easy to follow the authors’ logic. |  |  |
| • A major point of constructive criticism lies with one of the main tenets of the review: Specifically, that effective programs (those that results in sustained reduction of opioid use), necessitate a “change in the opioid prescriber.” Although the “realist” portion of the review is understandably fraught with challenges for those who rely upon rigorous statistical analyses to generate hypotheses, this seems like an incorrect and far too simplistic conclusion.  • Based on the authors’ summaries, this seems much more like an issue of expertise, rather than a simple change in opioid prescriber/provider.  • The statement that “…results from this review are quite clear that such programs will not be effective in reducing opioid use unless there is a change in the primary opioid prescriber” (starting at line 541) seems unfounded.  • The authors more/less acknowledge that their conclusion may be oversimplified. For example by stating that the change in opioid use may be explained by a different mechanism, such as the referral process itself (see lines 387-388). Nonetheless, the authors go on to explicitly say that primary care should input a mechanism for changing providers, as if what was found in the review was a collective result of a simplistic, literal change in the person prescribing the opioid.  • The most important change that accompanies a change in provider however, appears to be a vast increase in the expertise of the providers. From this reviewer’s perspective, that should be the primary point, rather than a simple change in providers. | Thank you for this very insightful comment. With respect to this activity of “changing the prescriber”, the data available from this review underdetermined this activity. Since most evaluations were conducted from the perspective of tertiary-level programs without reference to the primary care settings or primary prescriber, our ability to make inferences about the referral process itself was quite limited. Longitudinal evaluations from the patient perspective, rather than program perspective, would help to rectify this evidence gap. It would also help to rectify the noted evidence gap regarding long-term, post-program outcomes.  On reflection of this review comment, in all cases from the included literature where there was successful involvement from another prescribers, it could be inferred that such prescriber did have more expertise with respect to chronic pain management and opioid prescribing. As such, we have modified our discussion statements to reflect this. This does accord with the approach of shared-care which we had referenced in the manuscript. | Page 29, Lines 567-569 |
| • It would be helpful to have a table with all 8 synthesis statements. | Thank you for this suggestion. We have added a table with all synthesis statements. | Page 16, Lines 259-261 |
| • The right side of Table 1 is cut off. | Done in accordance to formatting guidelines for PLoS ONE (<https://journals.plos.org/plosone/s/tables>), the full table can be viewed in Word’s Draft View. |  |
| **Reviewer #3** | | |
| This is an excellent review and contribution to the literature of opioid use in patients with chronic pain. |  |  |
| Page 2 line 63 reference? Please place reference here | The reference for this is at the end of the following sentence (line 68). |  |
| Page 3 line 73-74, this sentence is unnecessarily accusatory. The opioid crisis is complicated it is not entirely iatrogenic. | Changed from “iatrogenic origin of” to “iatrogenic contribution to” to acknowledge that there are multiple contributing factors to these epidemics, an important component of which are iatrogenic | Page 3, Line 74 |
| Page 3, line 83-4, sentence is poorly written | Reworked the opening of the paragraph | Page 3, Lines 85-86 |
| Page 3 line 94: do not capitalize the W in What | Changed | Page 3, Line 96 |
| Table 1 did not fit on the page 8-13 | Done in accordance to formatting guidelines for PLOS ONE (<https://journals.plos.org/plosone/s/tables>), the full table can be viewed in Word’s Draft View. |  |
| Page w13: not sure that a program that ran for 1-5 days is in any way similar to longer running program. Please explain why this was included: | We did not use duration of program as an inclusion or exclusion criterion, and we anticipated, based on the findings Frank et al.’s systematic review, that there would be significant heterogeneity with respect to program duration, as well as a variety of other program characteristics. It is this heterogeneity and complexity that motivated us to take a realist review rather than traditional systematic review approach to synthesizing this literature.  Please also note the discussion on page 22 / lines 410-416 of the original submission regarding differences in program duration / intensity according to setting. |  |
| Page 14: Lines 229and 230. Confusing sentence | Thank you – we have clarified this sentence. | Page 15, Lines 246-247 |
| Page 15: Participants: We would expect different outcomes for patients with any form of opioid use disorder compared to those who are on chronic opioids for chronic pain. Please explasin why it is acceptable to lump them all together for the purposes of this study. | All included studies required the patient population to be people living with chronic pain and using opioid analgesics. Opioid use in the context of chronic pain can fall on a spectrum ranging from no aberrancy in use to clear opioid use disorder. Clinicians, especially in primary care settings where the majority of chronic pain care is delivered, treat people along this spectrum. Opioid use patterns can also be dynamic over time in a single individual. Given this diversity of use, and often clinical uncertainty about the nature of use, we felt it was important to consider the entire range of opioid use in the context of chronic pain. We did not note any marked difference in the structure and nature of programs that specifically focused on patients who had evidence of opioid use disorder. |  |
| Page 17, line 299: Please define MPRP | Added [multiprofessional rehabilitation programs] | Page 19, Line 320 |
| Page 18, line 327: define COT- | Added [chronic opioid therapy] | Page 20, Line 348 |
| Page 20-1, lines 376-378, “Some studies did report that higher proportions of non-completers were people on opioids…..” this is confusing, since the purported purpose of the this paper is to investigate the success of tapering opioids, why would the authors be looking at studies of patients who are not on opioids? | Some chronic pain management programs included a mix of patients who were on opioids and patients who were not on opioids. We have clarified this in the text. | Page 22, Lines 399-400 |
| Page 21, line 381 clarify opiates versus opioids | Removed opiates and changed to opioids. Thank you for identifying this – no distinction was intended. | Page 22, Line 404 |
| Page 23, lines 434-436 Did any of the programs look at preexisting opioid use disorder? | Added a sentence that none of the programs examined for OUD as a factor. | Page 25, Lines 461-462 |
| Page 29-30. Excessively and unnecessarily long | Given the established approach of realist reviews, we felt it was important to address in detail the underdetermination of theory by data available through this review process. We are not aware of others who have addressed, even briefly, the challenges of applying realist approaches to biomedical interventions and so feel that it important to provide some elaboration of this challenge. |  |

| **Journal requirement** | **Response** | **Location of edits** |
| --- | --- | --- |
| 1. Please ensure that your manuscript meets PLOS ONE's style requirements, including those for file naming. | Thank you – we have reviewed these style requirements and have ensured that all submitted documents conform accordingly |  |
| 2. Please report the date of last search, ensuring that it has been performed in order to allow the inclusion of studies published in the past 12 months. | We have included the dates of the last search for each component of our search strategy. Given that the review already covers 95 studies from 5 decades of literature and that there was consistency in our findings across these time periods and studies, we did not conduct a repeat formal literature search to capture studies from the last 12 months.  In line with the iterative development of realist analysis, we have conducted informal searches for new relevant literature and have not identified any additional studies that would substantively change our findings. | Page 5-6, Lines 140, 148, 150-155 |
| In addition, please discuss the quality and biases introduced by each of the included studies, or justify why this has not been performed. | Please see our existing comment regarding quality appraisal in realist reviews and associated reference in the **Strengths and limitations** section. | Page 30, Lines 588-594 |
