# Supplementary material for: Multidisciplinary care for opioid dose reduction in patients with chronic non-cancer pain: A systematic realist review
Source: PLoS One. 2020 Jul 27;15(7):e0236419. doi: 10.1371/journal.pone.0236419 (PMC7384622; doi:10.1371/journal.pone.0236419)
Supplement: Attachment — Submitted filename: MDC opioid response to reviewers.docx [file pone.0236419.s004.docx]

**Response to Reviewers**

Manuscript: *Multidisciplinary care for opioid dose reduction in patients with chronic non-cancer pain: A systematic realist review*

| **Reviewer Comment** | **Response** | **Location of edits** |
| --- | --- | --- |
| **Reviewer #1** | | |
| The authors did a thorough and detailed review of my comments and did an excellent job addressing my feedback and updating their manuscript. It is now a thoughtful and well constructed piece which will add nicely the literature regarding clinical opioid management. | Thank you for the thorough review of this manuscript. | N/A |
| **Reviewer #2** | | |
| Overall, the authors did a good job of addressing reviewers' comments.  However, I continue to think that Summary Statement #5 is overly simplistic. In the example included on page 23, while the occupational therapist and social worker may have been experienced, neither was a pain physician. In the second example on page 23, it is unclear when a “different provider” (from page 23: “…however, the opioid prescribing was taken over by the different family physician…”) took over. Together, these two examples do not convince the reader that simplistically changing a provider would significantly reduce opioid consumption.  Perhaps clarifying or using different examples would help make the point? | Thank you for the review and for identifying this concern.  We have clarified in the narrative preceding Summary Statement #6 that though the outcome between the two examples given was divergent (Seal 2017 resulted in opioid dose reduction and Angeles 2013 did not), these outcomes are both consistent with the need to change the prescriber to achieve reductions in opioid doses.  For the second example (ref 95, Seal 2017), we have clarified that the primary care physician with special expertise in chronic pain took over opioid prescribing at the time of referral to the Integrated Pain Team and also provided detailed recommendations back to the referring family physicians for long-term management. We hope this helps to clarify this process.  We have clarified in the first example (ref 28: Angeles 2013) that program participation required referral from the primary care physician. This program included an experienced occupational therapist and social worker (so was multidisciplinary, but without a physician), required referral by the primary care physician, resulted in some improvements in pain and health utilization, but *did not* result in any changes in opioid doses. Thus, we identified this is a good counterfactual example suggesting that without changing the prescriber (as was done in the example above, Seal 2017), opioid doses are unlikely to change.  As noted in our previous response and in the manuscript, since the vast majority of evaluations were based in tertiary care settings without reference to the primary care provider / primary opioid prescriber or referral process, there are no further examples for us to draw on to clarify this synthesis statement. However, we can have confidence in this statement since the majority of programs (as identified in synthesis statement #4) did involve active medication management, i.e. taking over prescribing from the previous prescriber during the patient’s participation in the program. In addition, we did not identify any contradicting examples.  It is important to note that our identified key program components (behaviour change, active medication management / changing the prescriber, and analgesic substitution) are described as interdependent - each are posited as necessary but none are independently sufficient in driving changes in opioid doses. We are not claiming that simply changing a *provider* would reduce opioid consumption, but that changing the *prescriber* is one essential component for multidisciplinary care programs to result in opioid dose reductions. If changing the pain provider was a necessary and sufficient condition, then we would expect all multidisciplinary care programs to result in opioid dose reductions – which is certainly not consistent with the included literature. Further empirical work making use of these findings will be essential in refining the set of necessary conditions and their relations with one another. | Page 23, lines 414-429 |
